# Lipidomics as a Diagnostic Tool for Prostate Cancer

**DOI:** 10.3390/cancers13092000

**Published:** 2021-04-21

**Authors:** Magdalena Buszewska-Forajta, Paweł Pomastowski, Fernanda Monedeiro, Justyna Walczak-Skierska, Marcin Markuszewski, Marcin Matuszewski, Michał J. Markuszewski, Bogusław Buszewski

**Affiliations:** 1 Department of Biopharmaceutics and Pharmacodynamics, Faculty of Pharmacy, Medical University of Gdańsk, Aleja Generała Józefa Hallera 107, 80-416 Gdańsk, Poland; michal.markuszewski@gumed.edu.pl; 2 Institute of Veterinary Medicine, Faculty of Biological and Veterinary Sciences, Nicolaus Copernicus University, 87-100 Toruń, Poland; 3 Department of Environmental Chemistry and Bioanalytics, Faculty of Chemistry, Nicolaus Copernicus University, 87-100 Toruń, Poland; pomastowski.pawel@gmail.com (P.P.); fernandamonedeiro@hotmail.com (F.M.); bbusz@umk.pl (B.B.); 4 Interdisciplinary Centre of Modern Technologies, Nicolaus Copernicus University, 87-100 Toruń, Poland; walczak-justyna@wp.pl; 5 Department of Urology, Faculty of Medicine, Medical University of Gdańsk, Smoluchowskiego 17, 80-214 Gdańsk, Poland; marcin.markuszewski@gumed.edu.pl (M.M.); marcin.matuszewski@gumed.edu.pl (M.M.)

**Keywords:** prostate tissue, metabolomics, prostate cancer, MALDI-ToF/MS, ESI-QqQ/MS, lipidomics, phospholipids

## Abstract

**Simple Summary:**

Prostate cancer (PCa) is one of the leading cancer deaths in man’s world. Due to the lack of a fast and sensitive diagnostic method, PCa is only recognized in the late stadium of the disease. For this purpose, new tools are sought for sensitive diagnosis. One of them is the use of mass spectrometry. The main goal of the study was to perform target lipidomic analysis of prostate tissue with the use of two imaging methods: matrix-assisted laser desorption ionization with time-of-flight mass spectrometer, and electrospray ionization with triple quadrupole mass spectrometer. Statistical evaluation provided the knowledge about the phospholipids alteration linked with PCa progression. In practice, its recognition enables development of a quick and sensitive diagnostic method. The acquired knowledge may also lead to the increasement of hormone therapies effectiveness.

**Abstract:**

The main goal of this study was to explore the phospholipid alterations associated with the development of prostate cancer (PCa) using two imaging methods: matrix-assisted laser desorption ionization with time-of-flight mass spectrometer (MALDI-TOF/MS), and electrospray ionization with triple quadrupole mass spectrometer (ESI-QqQ/MS). For this purpose, samples of PCa tissue (*n* = 40) were evaluated in comparison to the controls (*n* = 40). As a result, few classes of compounds, namely phosphatidylcholines (PCs), lysophosphatidylcholines (LPCs), sphingomyelins (SMs), and phosphatidylethanolamines (PEs), were determined. The obtained results were evaluated by univariate (Mann–Whitney U-test) and multivariate statistical analysis (principal component analysis, correlation analysis, volcano plot, artificial neural network, and random forest algorithm), in order to select the most discriminative features and to search for the relationships between the responses of these groups of substances, also in terms of the used analytical technique. Based on previous literature and our results, it can be assumed that PCa is linked with both the synthesis of fatty acids and lipid oxidation. Among the compounds, phospholipids, namely PC 16:0/16:1, PC 16:0/18:2, PC 18:0/22:5, PC 18:1/18:2, PC 18:1/20:0, PC 18:1/20:4, and SM d18:1/24:0, were assigned as metabolites with the best discriminative power for the tested groups. Based on the results, lipidomics can be found as alternative diagnostic tool for CaP diagnosis.

## 1. Introduction

The prostate is a small gland, the functionality of which is dependent on hormones, mainly androgens. In the physiological state, androgens are responsible for the cell growth and functional activities of this gland. By binding to the androgen receptor (AR), androgens stimulate the differentiation of luminal epithelial cells. The AR also plays a significant role in the functions of the prostate because it modulates the production of a crucial protein, namely prostate-specific antigen (PSA). Screening of PSA along with rectal examination is widely used in the diagnosis of PCa. However, due to the fact that PSA is organ-specific but not a characteristic of PCa, screening of this protein often leads to misdiagnosis [1]. For this reason, PCa diagnosis is proved by biopsy.

Recent research indicates the significant role of androgens and related receptors in enhanced lipogenesis in PCa [1]. In the process of cancerogenesis, androgens affect cell proliferation, differentiate the secretory function of cells, and induce the formation of cytoplasmic lipid droplets [2]. Moreover, AR has been recognized as a key player in the development of PCa because it modulates the expression of several lipogenic enzymes, in both primary and metastatic tumors. This observation inspired several researchers to study the influence of lipids in carcinogenesis. The role of lipids in PCa development can be more important than it was considered because of their physicochemical properties and varying biological roles, and hence, they may be crucial in the diagnosis and treatment of PCa. Lipids are the main building blocks of cell structure. They are used as a major source of energy and take part in signaling. Few publications have proposed the possible mechanisms of cancerogenesis, involving alterations in oxidation, glycolysis, and tricarboxylic acid cycle. However, there is still a lack of knowledge about how lipids influence the development of PCa [2,3,4,5]. For this reason, it is important to understand the pathogenesis of PCa. Additionally, epidemiological studies suggest that fat consumption and supplementation may have a significant impact on the development and progression of PCa [6]. Thus, recognizing the contribution of lipids in tumor development may be crucial in the diagnosis and treatment of this disease.

Undoubtedly, highly proliferative cancer cells have a higher demand for lipids. Suburu pointed out that metabolic shift from catabolic to anabolic processes is a hallmark of cancer cells [4]. In the case of PCa, upregulation of fatty acids, cholesterol, phospholipids, and androgens may be observed as a consequence of the overexpression of fatty acid synthase (FAS) or extracellular lipolysis of triglycerides [2,3]. Contrary to normal somatic cells, cancer cells use de novo synthesis of fatty acids for signaling, for building the cellular membrane, or for energy supply [7].From the clinical point of view, overexpression of FAS enhances the invasion ability of cancer cells [7]. Several studies have indicated phospholipids as important disease predictors. Ramirez de Molina et al. [8] showed the increased concentration of two major structural phospholipids, namely phosphatidylcholine (PC) and phosphatidylethanolamine (PE), as crucial membrane components in rapidly proliferating cancer cells. This was due to the enhanced expression and activity of choline kinase, found as an oncogenic factor [8,9]. Lipidomics is one of the analytical tools that can be successfully used to identify, quantify, and finally understand the structure and function of lipids associated with cancer development. However, due to the high diversity in a structure (headgroup or location 1’ of double bonds, as well as the linkage between the functional groups in the molecule), determination of all groups of lipids is impossible with the use of one universal method [10]. For this reason, mass spectrometry (MS) is widely applied in lipidomics, as it can characterize the analytes by their mass-to-charge ratios (*m*/*z*) without the need for a sample separation step. In addition, analysis with MS allows obtaining information about the structure, by the study of fragmentation reaction. Furthermore, MS is characterized by high selectivity and sensitivity. However, its application requires the ionization of analytes. The most popular methods used for ionization are atmospheric pressure chemical ionization [11] and electrospray ionization (ESI) [12].

Nowadays, there is growing interest in the use of the matrix-assisted laser desorption and ionization (MALDI) method due to its high sensitivity (few attomoles of an analyte is sufficient) and ease of use. Although MALDI-MS is widely applied in proteomics and genomics, its use in lipidomics is yet to be investigated [13].

The main aim of this study was to explore the lipidomic signature of PCa. A target lipidomic analysis was performed using two complementary analytical techniques, namely MALDI-ToF/MS and ESI-QqQ/MS, to identify the phospholipids that may potentially serve as prognostic and/or early diagnostic markers of PCa. For this purpose, 80 fresh prostate tissue samples were collected from patients with PCa (*n* = 40) and non-cancers (*n* = 40). The obtained results were analyzed using multivariate statistical analysis and chemometric method to select the lipidomic metabolites with a greater ability to discriminate between the controls and PCa cases.

## 2. Materials and Methods

### 2.1. Biological Material

All the participants enrolled in the study were selected on the basis of an examination carried out at the Department of Urology at Medical University of Gdańsk and were matched according to sex and age (*p* = 0.30). The mean age of the PCa patients was 67.5 ± 5.5 years, and that of the controls was 68.1 ± 6.46 years. The participants were divided into two groups: patients with diagnosed PCa *(n* = 40) and non-cancer controls (*n* = 40). The studied group consisted of samples obtained from patients with diagnosed PCa. The diagnosis was done on the basis of elevated PSA level during screening tests, digital rectal examination, ultrasound examination, and histopathological evaluation. The mean PSA level was 14.80 ± 12. ng/mL, where maximum concentration was 45.53 ng/mL and minimum 0.008 ng/mL. Tissue samples were evaluated according to a histopathological protocol and Gleason grading. The Gleason grade was ranging from 6 to 10. Among them, 11 cases were assigned by Gleason grade 6, 14 corresponded to Gleason grade 7, 4 described by Gleason grade 8, 10 cases assigned as Gleason grade 9, and 1 case described by Gleason grade 10. The characteristics according to the Gleason score are listed in Table 1. The control group was consisted of samples obtained from patients with diagnosed benign prostatic hyperplasia. The mean PSA level in non-cancer controls was 6.34 ± 3.82 ng/mL, with minimum value 0.868 ng/mL and maximum 18 ng/mL.

Exclusion criteria included treatment with hormones, metastasis to other organs, and presence of diabetes or any other chronic diseases. The study was conducted in accordance with Independent Commission for Bioethics Research, Medical University of Gdańsk (NKBBN/432-119/2017). All the subjects gave written consent to participate.

### 2.2. Chemicals

Water LC–MS Chromasolv, ethanol, acetonitrile, trifluoroacetic acid, formic acid, and isopropanol were obtained from Sigma Aldrich (Steinheim, Germany). Chloroform was purchased from POCH (Poland), while ultrapure water was obtained with the Milli-Q water system purchased from Millipore (Bedford, MA, USA). All chemicals needed for the MALDI-MS analysis (with the highest available purity) were purchased from Fluka Feinchemikalien GmbH (part of Sigma Aldrich). α-Cyano-4-hydroxycinnamic acid (HCCA) was obtained from Sigma Aldrich (St. Louis, MO, USA) and used as a matrix for the MALDI analysis. Sample decomposition was carried out using polished steel targets (Bruker Daltonik). External calibration was performed using protein Calibration Standards I (Bruker Daltonik, Bremen, Germany). Standards of L-phosphatidylcholine (≥99%) (PC), L-phosphatidylethanolamine (≥98%) (PE), sphingomyelin (SM) (≥95%), L-lysophosphatidylcholine (≥98%) (LPC), L-lysophosphatidylethanolamine (≥97%) (LPE), L-phosphatidylglycerol (≥98%) (PG), L-lysophosphatidylglycerol (≥98%) (LPG), L-phosphatidylinositol (≥98%) (PI), L-lysophosphatidylinositol (≥98%) (LPI), phosphatidic acid (≥98%) (PA), and lysophosphatidic acid (≥98%) (LPA) from egg yolk were purchased from Larodan Lipids (Malmö, Sweden).

### 2.3. Sample Preparation

#### 2.3.1. Preparation of Chloroform:Methanol Solution (1:2; *v*/*v*)

The extraction solution was prepared by adding 150 mL of chloroform to 300 mL of methanol in a volumetric glass. The solution was vortex-mixed.

#### 2.3.2. Extraction Procedure for Tissue Samples

Each tissue sample was weighed and transferred into a separate tube. According to the weight of tissue, an appropriate volume of the prepared extraction solution was added; for each 10 mg of tissue, 200 μL of chloroform:methanol solution was added. Each sample was homogenized using a Bullet Blender Storm homogenizer (Next Advance, New York, NY, USA). Sample homogenization involved three cycles, with each cycle carried out for 1 min, followed by cooling in ice for 1 min. In the last step, 400 μL of pure water was added to 1 mg of the tissue sample, and the three cycles of homogenization were repeated again. Finally, the tubes containing samples were placed in a water bath and sonication was carried out (15 min, room temperature). Then, the samples were allowed to rest for 15 min to obtain two non-mixed phases. Each organic phase (bottom layer) was collected and transferred to a new tube. The extracts were evaporated to dryness using a vacuum concentrator (CentriVap DNA Vacuum Concentrators; Labconco, Kansas City, MO, USA) for 2 h at 36 °C. The dry residue was dissolved in 200 μL of methanol.

### 2.4. Instrumentation and Lipidomic Profiling

#### 2.4.1. MALDI-ToF/MS Analysis

In the lipidomic analysis, the classical “dry droplet” method was used [14]. The protocol involved direct spotting of a sample onto a MALDI target. For this purpose, lipidomic extract of biological samples were first spotted on the surface of a polished steel target. Then, the sample spots were covered with 1 µL of the HCCA matrix solution. Finally, the spots were air-dried before the analysis. Each sample was smeared onto MALDI targets in triplicate. All spectra were acquired on an ultrafleXtreme MALDI-ToF/ToF mass spectrometer system (Bruker Daltonik, Bremen, Germany). The mass spectra were calibrated using the cesium triiodide cluster [14]. This system includes modified Nd:YAG laser (Smartbeam IITM) operating at a wavelength of 355 nm and a frequency of 2 kHz. Analyses were carried out in linear positive mode at an acceleration voltage of 25 kV. Spectra were recorded manually in the *m*/*z* range of 150–2000 with flexControl and flexAnalysis software. Each sample was analyzed in triplicate.

#### 2.4.2. ESI-QqQ/MS Analysis

The ESI-MS analyses were carried out using a Shimadzu ESI-MS/MS 8050 system (Tokyo, Japan), consisting of a binary solvent delivery system (LC-30 AD), a controller (CBM 20A), and an autosampler (SIL-30A). For instrument control, data acquisition, and processing, LabSolution 5.8 software was used. Analysis was carried out using isocratic solvent program. The composition of the mobile phase was as follows: 90% MeOH and 10% of 0.1% HCOOH solution. The solvent flow was set at 0.2 mL /min. The optimal parameters of MS were as follows: nebulizing gas flow—3.0 L/ min, heating gas flow—10 L/min, and temperature of the drying gas—250 °C. The interface temperature was set at 300 °C, while the heat block temperature was set at 400 °C. For the quantification of phospholipids, species were monitored in the scheduled multiple reaction monitoring mode. The total dwell time was 1 s. MRM analysis for identification of phospholipids are listed in Table S1 of Supplementary Materials.

### 2.5. Statistical Analysis and Chemometrics

Unsupervised and supervised multivariate methods were carried out. The primary database was normalized by the weight of tissue and consisted of analytical responses from 57 and 33 compounds detected by ESI-QqQ/MS and MALDI-ToF/MS analysis, respectively. Discriminant features were determined by applying Mann–Whitney U-test, using IBM SPSS Statistics v.23 software. Further data analysis was performed in R environment. Principal component analysis (PCA) was conducted using “prcomp” R function. For PCA, it was considered a matrix displaying detected metabolites (variables) and their respective abundances for each of the analyzed samples. The statistics were performed separately for matrices containing only results on MALDI-MS or ESI-MS observations. Only data presenting at least 10% of total abundance was considered; prior to analysis, the data was scaled by subtracting variable’s mean and dividing it by the standard deviation. PCA allows visualizing the general trends of data as well as providing information about the potential outliers [15]. Compound identification enabled the determination of four main groups of analytes, namely PCs, LPCs, SMs, and PEs. To search for the relationships between the responses of these groups of substances, also in terms of the used analytical technique, a correlation analysis was performed. Correlation plots were built using “corrplot” package, and the Spearman rank coefficient was selected as a correlation criterion. Volcano plots were created using “EnhancedVolcano” package, and *p* < 0.05 was set as the relevance criterion. The applied statistical methods provided information about the differences in the level of metabolites between the two tested groups and the existing intercorrelations.

In the next step, machine learning models were developed to discriminate the studied classes of samples (control and PCa). For this purpose, artificial neural network (ANN), as well as random forest (RF) algorithm was employed, applying “neuralnet” and “randomForest” R packages. For ANN, the following parameters were applied: number of neurons/hidden layers = 10, algorithm = “rprop+” (default, resilient backpropagation with weight backtracking), differentiable function = “logistic” (logistic/sigmoid function). The number of neurons in the input layer was 14 (number of variables that were selected for the model, refer to Section 3.1.4). The neurons constituting the output layer were two (number of assumed test categories, detailed below in this section). The performed ANN analysis was conducted in 99 and 228 steps (training epochs) and obtained errors were 0.53 and 5.97, for MALDI-MS and ESI-MS data, respectively. For RF, the following parameters were applied: number of trees = 2000, number of variables randomly sampled as candidates at each split = 8, cut-off = 1/k (majority vote wins, where k is the number of classes, i.e., 2).

Variable importance was verified based on the calculated mean decrease in accuracy [16] (in the case of RF) and Olden’s method output [17] (in the case of ANN). The data was split randomly into two groups: one half of the data (50%) comprised the training set (used for model learning), while the other half was used for testing (the previously developed model was used to make predictions on this new data; the observed rates of false positive, false negative, true positive, and true negative were used to evaluate performance by class). Ultimately, 10-fold cross-validation was conducted to obtain the mean accuracy of the final models (validation step); in this step, 95% of the dataset was used as training set while the remaining 5% were ascribed to the test set. Confusion matrices and receiver operating characteristic (ROC) curves were created to assess the performance of the classifiers, with “caret” and “ROCR” packages. ROC curves considered as input the probabilities computed by the model of a case to be labeled as belonging to a specific class, in addition to the actual classification values (1 for the state variable, 0 for control cases). Sensitivity, specificity, and overall model accuracy were provided by examination of confusion matrix.

## 3. Results

Lipid analysis was performed using MALDI-ToF/MS and ESI-QqQ/MS in both positive and negative ionization mode. MALDI-ToF/MS enabled to identify phospholipids containing choline (PC, LPC, SM) and ethanolamine (PE, LPE), while phosphatidylinositol (PI), phosphatidylserine (PS), phosphatidylglycerol (PG), and PA were identified by ESI-QqQ/MS.

**Figure 1:**
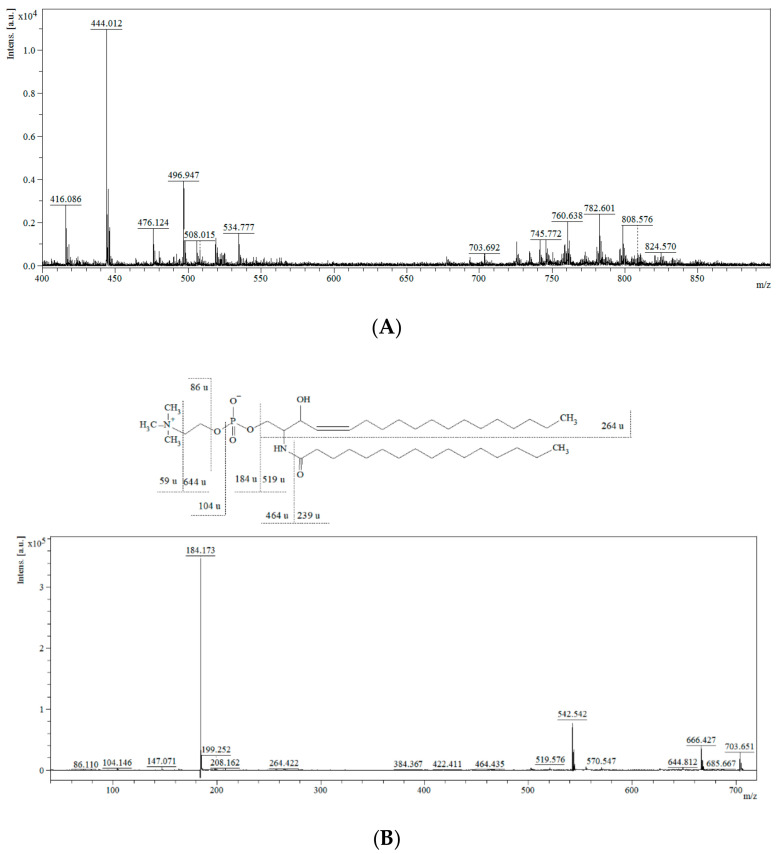
Mass spectra of the lipidomic profile obtained from MALDI-ToF/MS in positive ion mode (**A**) and MS/MS spectra and fragmentation pathway of SM (d18:1/16:0) *m*/*z* 703.651 (**B**).

**Figure 2:**
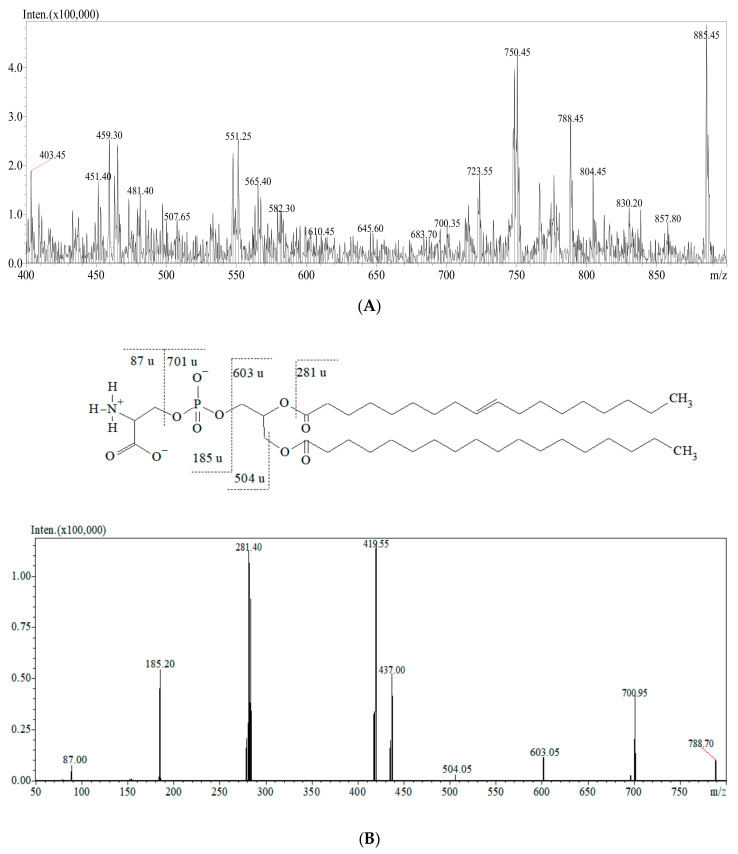
Mass spectra of the lipidomic profile obtained from ESI-QqQ/MS in negative ion mode (**A**) and MS/MS spectra and fragmentation pathway of PS *m*/*z* 788.70 (**B**).

The representative mass spectra of tissue lipidomic fingerprints obtained from the MALDI-ToF/MS and ESI-QqQ/MS analysis are presented in Figure 1 and Figure 2. Comparing the mass spectra, it can be observed that both the MALDI-ToF/MS and ESI-QqQ/MS techniques gave complementary information on lipids. Figure 1A presents the full scan in the positive ionization mode for the MALDI-ToF/MS analysis, and Figure 2A shows the full scan in the negative ionization mode for ESI-QqQ/MS. Moreover, tandem MS analysis was performed for each compound to obtain the fragmentation spectra. Exemplary fragmentation spectra as well as the probable fragmentation pathway of SM (d18:1/16:0) and PS are presented in Figure 1B and Figure 2B, respectively. SMs are phospholipids containing ceramide and often also choline. The *m*/*z* 703.651 represents a protonated molecular ion [M+H]^+^. The ion detected at *m*/*z* 644.812 corresponds to the neutral loss of trimethylamine [M+H-N(CH_3_)_3_]^+^ (59 u). The fragments detected at *m*/*z* 86.110, 104.146, and 184.173 correspond to “dehydrocholine” [C_5_H_12_N]^+^, choline [C_5_H_13_NO+H]^+^, and “head of PC” [C_5_H_14_NPO_4_+H]^+^, respectively. The fragments observed at *m*/*z* 264.422 correspond to a dehydrated long-chain base (18:1 chain), which is characteristic of the SM structure. The elimination of 464.435 u from the *m*/*z* 703.651 gave rise to an ion at *m*/*z* 239 corresponding to palmitic acid residue.

PS was identified in the negative ion mode. The ions at *m*/*z* 700.95 and 603.05 resulted from the neutral losses of a serine head group [C_3_H_5_NO_2_]^−^ (87.0 u) and phosphoserine moiety [C_3_H_8_NPO_6_]^−^ (185.2 u), respectively. The peak at *m*/*z* 281.40 corresponds to the neutral loss of oleic acid (C18:1). The fragment detected at *m*/*z* 504.05 corresponds to the loss of stearic acid [M-R_1_COOH]^−^. The ion at *m*/*z* 419.55 probably resulted from the neutral loss of oleic acid with a serine head group.

### 3.1. Chemometrics and Statistical Validation

#### 3.1.1. Principal Component Analysis

**Figure 3:**
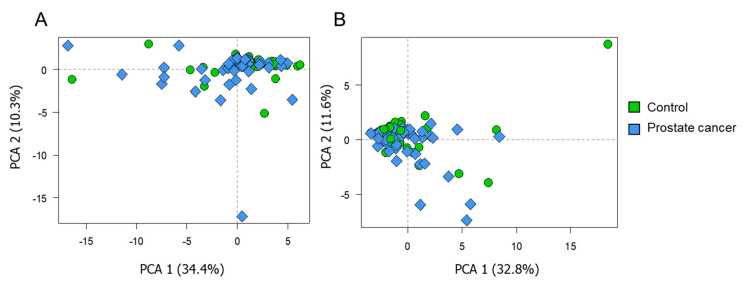
PCA score plots built on the data from ESI-QqQ/MS (**A**) and MALDI-ToF/MS (**B**) analysis. Diamonds and circles correspond to the profiles belonging to the PCa patients and control group, respectively.

The PCA plots are presented in Figure 3. Figure 3A corresponds to the data obtained with the ESI-QqQ/MS approach, while the PCA model shown in Figure 3B was built on the data obtained using the MALDI-ToF/MS technique. Samples are dispersed on the plot, based on their biological variability. The presented general trends did not vary with respect to the used technique. For both ESI-MS and MALDI-MS data, the first two components were able to describe around 44% of the total variance. Considering PCs 3 and 4 (not shown), these explained the following proportions of total variance: 8.38% and 5.76% (for ESI-MS data), 8.72% and 5.97% (for MALDI-MS data), respectively; In this sense, it was verified that projections including other principal components were still not able to improve the visualization of trends according to sample groups. In general, formation of isolated clusters according to the group of samples was not observed, which indicates that the total variance within the dataset is mainly attributed to various factors (age, tumor staging, intake of medicines, diet, inter-variability of metabolism, etc.). The absence of spontaneous distribution among the studied profile classes indicates that supervised statistical methods may be required to discriminate the profiles.

#### 3.1.2. Correlation Analysis

**Figure 4:**
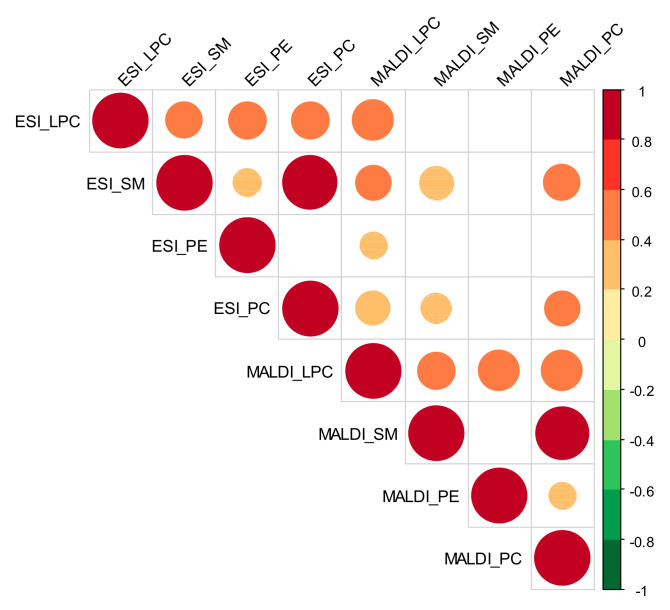
Correlation matrix built on the data using total response of phospholipids, in the case of data acquired from both techniques (MALDI: MALDI-ToF/MS analysis, ESI: ESI-QqQ/MS analysis). Color code refers to rho coefficient; the size of circles is proportional to the strength of correlations; blank cells correspond to nonsignificant bicorrelations (*p* > 0.05).

Correlation analysis was carried out using the total signals of phospholipids (summed up responses, per class of analyte). This approach took into consideration the corresponding 29 metabolites that were detected by both assays (MALDI-MS and ESI MS), namely: PE 7:0/7:0, LPC 16:1, LPC 16:0, LPC 17:1, LPC 17:0, LPC 18:3, LPC 18:2, LPC 18:1, LPC 18:0, SM d18:1/16:1, SM d18:1/16:0, PC 16:0/14:1, SM d18:1/18:3, PC 16:0/16:1, PC 16:0/16:0, PC 16:1/18:2, PC 16:0/18:2, PC 16:0/18:1, PC 18:2/18:3, PC 16:0/20:4, PC 18:1/18:2, PC 18:1/18:1, SM d18:1/22:0, PC 18:0/18:1, PE 20:1/20:4, PC 17:0/20:4, PC 16:0/22:6, PC 18:1/20:4, and PC 18:1/20:3. Figure 4 shows that a positive moderation correlation (Spearman coefficient, rho > 0.4) was detected between the total LPC and total SM/PE/PC signals, for both the analytical techniques used. In both cases, a strong positive correlation (rho > 0.9) was found between the total SM and PC. However, it should be noticed that no relevant correlation (empty squares) or a weaker correlation (rho < 0.4) was noted between most of the data obtained using the two different techniques. Negative correlations were also not observed.

#### 3.1.3. Assessment of Discriminating Variables

**Figure 5:**
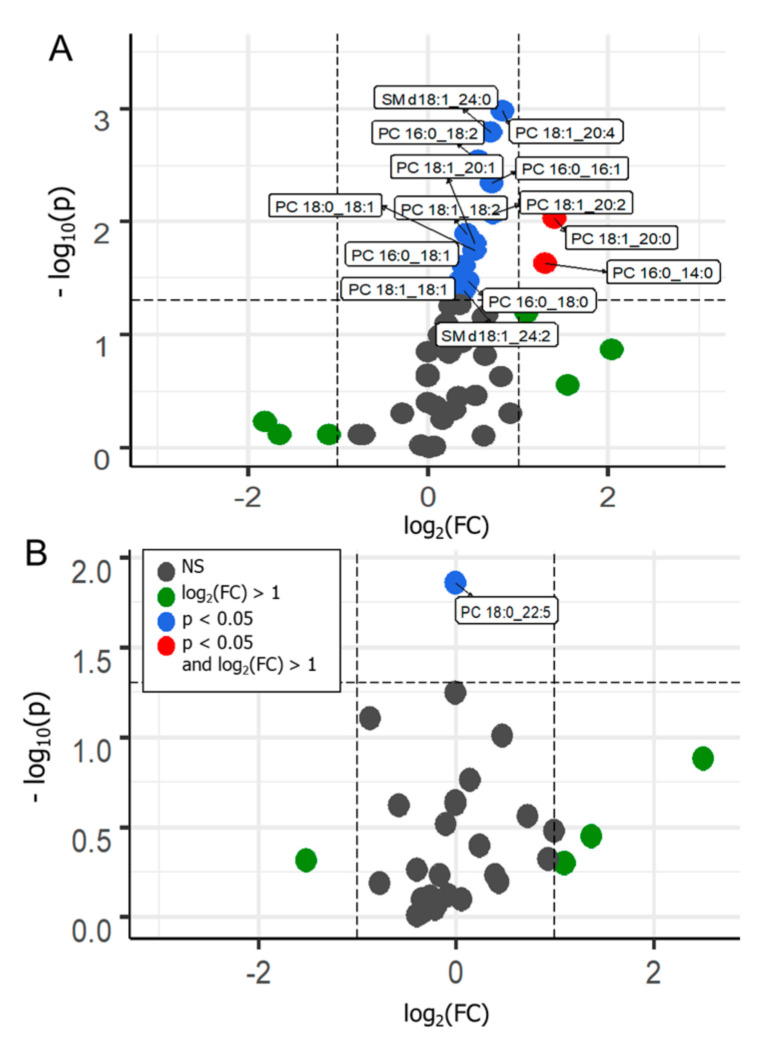
Volcano plots prepared from the data acquired from the ESI-QqQ/MS (**A**) and MALDI-ToF/MS (**B**) analysis, displaying the comparison between PCa vs. control cases. NS-nonsignificant; FC-fold change; significance threshold, *p* < 0.05.

Volcano plots (Figure 5) were used for supervised data inspection, to select the metabolites characterized by the most discriminating power. This type of plot allows combined visualization of fold change in the analyte’s response and its significance; the first one is expressed in terms of log_2_ of analyte intensity in the positive group divided by its intensity in the control group, and the latter is expressed in terms of −log_10_ of *p* value.

In a basic approach, metabolites placed above the threshold limit (horizontal dashed line) can be considered as distinguishing features. Compounds displayed on the left or right side of the graph correspond to those with increased or decreased responses in the positive cases, respectively. Compounds placed toward the top of the graph are those that displayed a greater significance as discriminant variables. Variables differentiating the two tested groups are indicated by blue and red colors in the graphs. Blue dots correspond to statistically significant variables (*p* < 0.05), while red ones refer to statistically significant variables with a fold change greater than 1.

Considering the cancer cases against the controls, in the data obtained from the MALDI-ToF/MS analysis, only one variable out of 33, namely PC 18:0/22:5, was indicated as a discriminant. The PC 18:0/22:5 level was downregulated in reference to controls. In the case of data obtained using the ESI-QqQ/MS analysis, 14 variables out of 57 were found as statistically significant. Among them, PCs and SMs can be noticed. All the indicated metabolites, PC 16:0/14:0, PC 16:0/16:1, PC 16:0/18:2, PC 16:0/18:1, PC 16:0/18:0, PC 18:1/18:2, PC 18:1/18:1, PC 18:0/18:1, PC 18:1/20:4, SM d18:1/24:2, PC 18:1/20:2, PC 18:1/20:1, SM d18:1/24:0, and PC 18:1/20:0, appeared to be upregulated in the positive samples. These results suggest that, for both types of analysis, latent metabolic patterns may be associated with the PCa cases. Therefore, supervised statistical models may allow discrimination between clinical conditions based on selected metabolites from the analyzed lipidome.

#### 3.1.4. Machine Learning Predictive Models

**Figure 6:**
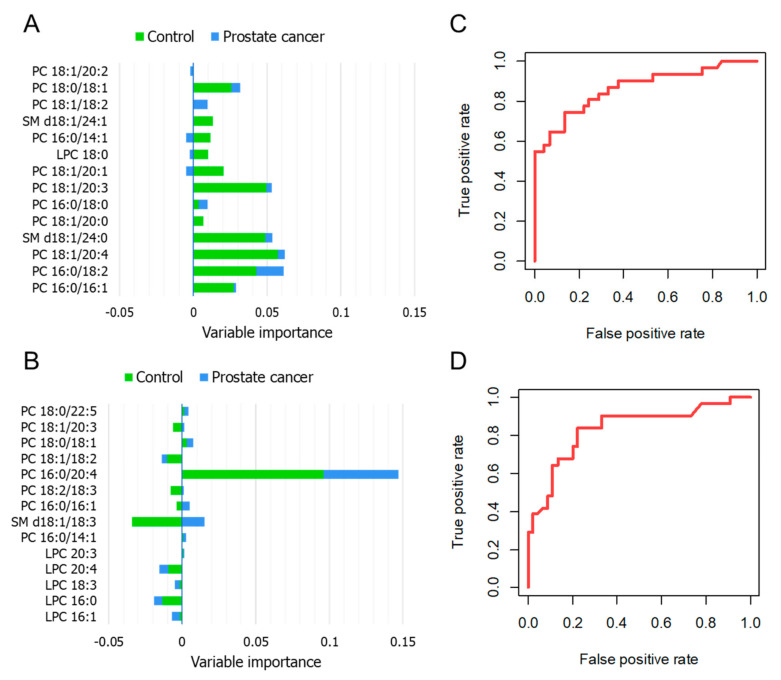
RF analysis—variable importance plot referring to the 14 most relevant features selected from the ESI-QqQ/MS (**A**) and MALDI-ToF/MS (**B**) analysis; ROC curves corresponding to the plotted probabilities calculated from the developed model for the ESI-QqQ/MS (**C**) and MALDI-ToF/MS (**D**) data, when PCa is labeled as the state variable.

Statistical models enabling the prediction of the studied conditions were developed. The RF approach is used for the examination of latent patterns and is based on the generation of multiple decision trees from independent training sets. The combined outputs fit the best solution for the attempted prediction [18]. In the first step, all the variables were considered for generating a preliminary model, and then the importance of variables was evaluated in terms of the calculated mean decrease in final accuracy if that specific feature would be removed from the system. The number of selected features to be incorporated in the final model should be as low as possible, without hindering the system’s accuracy. In this manner, 14 features ranked as the most relevant were chosen. Figure 6A,B present the metabolites selected from the ESI-QqQ/MS and MALDI-ToF/MS data, respectively, and their contribution to an output corresponding to the classes control or PCa (green and blue bars, respectively). For the ESI-QqQ/MS analysis, PC 18:1/18:2, PC 16:0/14:1, and PC 18:1/20:1 were found to be notably valuable in the discrimination between the investigated classes; the first one was positively correlated to the cancer group, the others were mostly positively correlated to controls and negatively correlated to PCa. In the MALDI-ToF/MS data, SM d18:1/18:3 was identified as the variable displaying greater decision-making power, mainly related to the PCa class. The ROC curves were built from the calculated probabilities derived from the developed classifiers (Figure 6C,D). The obtained area under the curve indicated that both datasets showed adequate classification performance, with the ESI-MS data providing a superior accuracy. The sensitivity and specificity of the proposed RF models ranged from 64.5 to 74.2% and from 84.5 to 89.0%, respectively. Table 2 summarizes the parameters related to the suitability of the prepared models.

**Figure 7:**
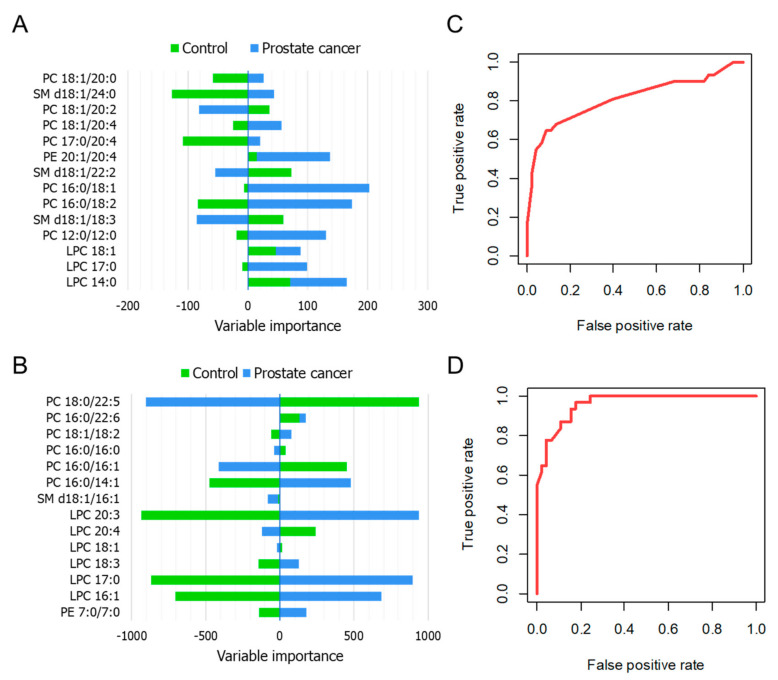
ANN analysis—variable importance plot referring to the 14 most relevant features selected from the ESI-QqQ/MS (**A**) and MALDI-ToF/MS (**B**) analysis; ROC curves corresponding to the plotted probabilities calculated from the developed model for the ESI-QqQ/MS (**C**) and MALDI-ToF/MS (**D**) data, when PCa is labeled as the state variable.

The ANN algorithm is inspired by the neurons of the brain, where a given input elicits responses represented by the interlayer connection weights, which influence to a lesser or greater extent a certain outcome (ANN output) [19]. As previously described, a preliminary model was built considering all the available variables, and from these, 14 were selected based on their importance in terms of combined layer weights culminating in a certain output (Olden’s method). Figure 7A,B present the variable importance plots displaying the chosen features. In the case of the ESI-QqQ/MS data, PC 16:0/18:1, PC 16:0/18:2, and PC 12:0/12:0 were presented as particularly valuable variables; all of these were positively correlated with the cancer group and negatively correlated with the control cohort. Regarding the results of the MALDI-ToF/MS analysis, PC 16:0/14:1, LPC 20:3, LPC 17:0, LPC 16:1, and PE 7:0/7:0 appeared to be specifically related to the PCa samples, while showing a negative association with controls. The ANN algorithm applied to the ESI-QqQ/MS and MALDI-ToF/MS results showed an accuracy of 72.5 and 87.5% in the validation step. The ROC curves (Figure 7C,D) demonstrated that the sensitivity and specificity of the proposed ANN classifier ranged from 82.2 to 91.1% and from 64.5 to 96.8%, respectively. When considering adequacy of sensitivity and specificity parameters, the ANN model was demonstrated to be more suitable for the available data than the RF classifier. For ANN, it can be observed that MALDI-MS data as input provided even superior value of overall model accuracy in the validation phase. Considering that the used data possibly contain several relevant nonlinear relationships (please refer to Section 2.5), it can be expected that the ANN algorithm will perform better than RF once it is reported that this first model has superior ability to solve nonlinear problems and deal with noisy data.

## 4. Discussion

Tissue is considered to be a quite difficult biological matrix. Collection of tissue is invasive and problematic due to ethical reasons. Although, the tissue matrix is the most informative one and a proper histopathological assessment coupled with lipidomic study may reveal the complex signature of the changes occurring during carcinogenesis and disease progression. From the histopathological point of view, tumor progression is associated with morphological changes. Undoubtedly, alterations in the tissue morphology and composition of the cell membrane may affect the transport system, cellular tightness, and the activity of enzymes present in the membrane. All these may be crucial for cell proliferation and growth contributing to the progression of PCa [5].

Previous lipidomic studies have determined the molecular mechanism underlying PCa development. Increased levels of fatty acids in tumors may be due to the increased expression of the biosynthetic genes and may result from the upregulated conversion of glucose and/or glutamine into cytosolic acetyl-CoA. Taking this into consideration, it is reasonable to assume that the production of phospholipids is accelerated during carcinogenesis [20]. The level of phospholipids is strictly associated with the clinical parameters of PCa (clinical stadium, pathological grade, and Gleason score) [5]. It has been hypothesized that the total increase in the level of phospholipids is linked with further increased progression of PCa and the intensified need for phospholipids during fast cell proliferation which is a characteristic of carcinogenesis [5]. The abovementioned mechanism is one of the possible metabolic pathways in tumor development. Suburu et al. [4] pointed out that metabolic shift from catabolic to anabolic processes is a hallmark of cancer cells. Oxidative phosphorylation is replaced by glycolysis which delivers the basic precursors for the synthesis of membrane, protein, and nucleic acid [20]. Furthermore, this hypothesis was confirmed by the study conducted on cell culture by [21].

Neoplasm microenvironment seems to be sensitive to alterations in fatty acid synthesis pathways, as these provide resources that are essential for tumor progression. In malignancies, factors such as altered availability of substrates, differential gene expression, and changes in enzymatic activity are potentially connected with variations in the levels of PCs. Moreover, biosynthesis and catabolism of PCs may be associated with the processes related to cell survival, proliferation, and programmed death, which highlight the role of PCs and their derivatives in the key mechanisms involved in cancer development [20].

In the proposed study, two lipidomic approaches, namely MALDI-TOF/MS and ESI-QqQ/MS were used in order to confirm the hypothesis regarding changes in lipidomic signature within cancerogenesis.

The innovative model for searching of potential prostate cancer biomarkers supported by advanced chemometric analysis was proposed. By definition, the ionizing MALDI and ESI in a broad spectrum of specificities are complementary to each other. However, the MALDI-TOF/MS technique allows for a global imaging of the current physiological state of the organism at the molecular level. Undoubtedly the main advantage of this method is the ability to monitor changes which occur for a whole panel of metabolites rather than for individual ones. Moreover, this method requires small amount of sample and relatively simple sample preparation protocol. Additionally, MALDI-TOF/MS can be characterized by high specificity and the possibility for process automation. MALDI-TOF/MS is commonly used in another diagnostic area in the field of bacteria identification (biotyper) or sequencing of protein.

The second method used within the project is ESI-QqQ/MS. Semiquantitative analysis followed by the advanced chemometric analysis is enabled to select metabolites assigned as the most discriminative for tested groups. This analytical approach can be found as a comprehensive method which provides information about metabolic changes occurring during carcinogenesis.

**Figure 8:**
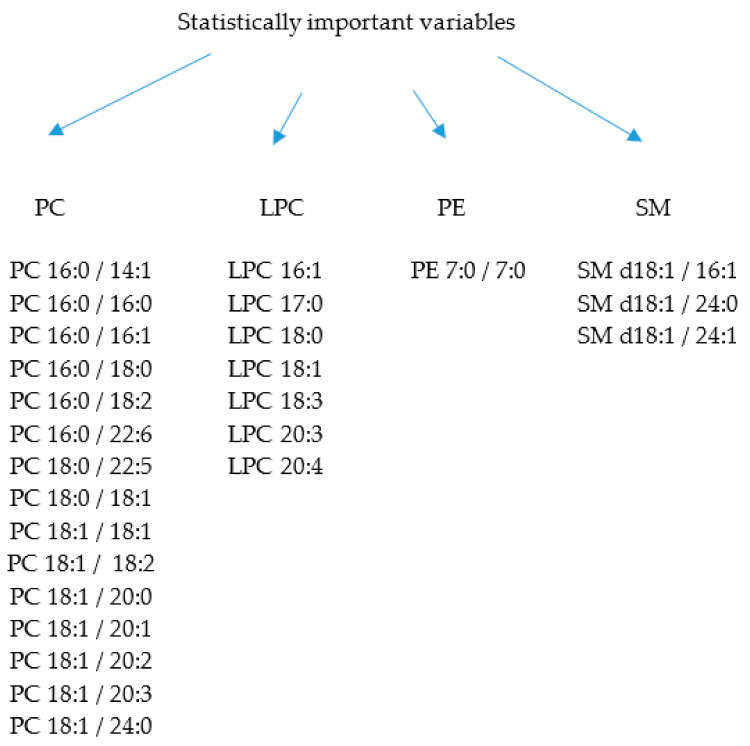
Variables assigned as statistically significant for PCa development.

In this study, the application of lipidomic analysis enabled us to explore the alterations in the synthesis and metabolism of phospholipids. A total of 26 phospholipids were selected with the use of the chemometric approach. Among them, four subclasses, namely PC (15 metabolites), LPC (7 metabolites), PE (1 metabolite), and SM (3 metabolites), can be marked (Figure 8). It is worthy to mention that the use of univariate methods to assess the discriminating power of metabolites is a straightforward approach but may not explain the collective contribution of variables to a specific condition. An alternative relies on the combination of statistical techniques, to better represent relevant relationships between variables and to provide a scheme of how condition specification is given by these features [22]. Nevertheless, the strategy here presented has exploratory value, being useful to indicate possible discriminating features that can be further investigated and submitted to experimental validation procedures in order to confirm their status as potential biomarkers.

A detailed description of statistically significant metabolites determined by the acknowledge method is shown in Table 3. As can be observed, only 7 out of 29 phospholipids (over 24%) were selected based on each of the applied statistical methods. Among them, PC 16:0/16:1, PC 16:0/18:2, PC 18:0/22:5, PC 18:1/18:2, PC 18:1/20:0, PC 18:1/20:4, and SM d18:1/24:0 were assigned as metabolites linked with PCa development, according to the three used statistical approaches. Further, 10 of the phospholipids (34.5%) were selected using two out of three statistical methods. These included LPC 16:1, LPC 18:3, LPC 20:3, LPC 20:4, PC 16:0/14:1, PC 16:0/18:0, PC 16:0/18:1, PC 18:0/18:1, PC 18:1/20:1, and PC 18:1/20:2.

**Figure 9:**
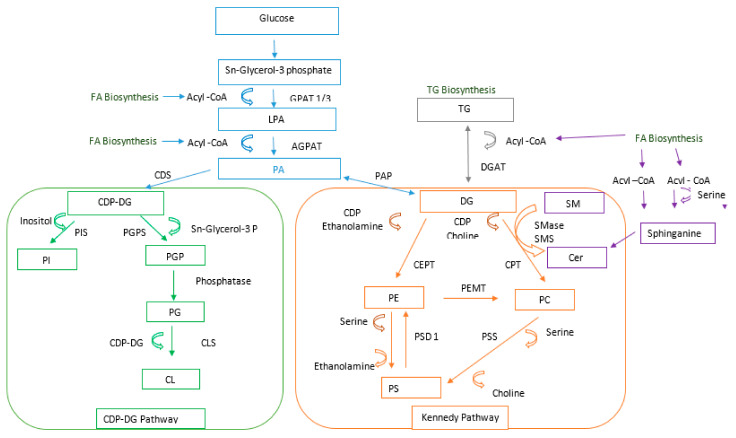
Phospholipids synthesis and modeling pathways. Acyl Co A—acyl coenzyme A; FA—fatty acid; GPAT 1/3—glycerol-3-phosphate acyltransferase 1/3; LPA—lysophosphatidic acid; APGAT 1—acylglycerol-3-phosphate O-acyltransferase 1; PA—phosphatidic acid; CDS—cytidylyltransferase; CDP-DG—cytidine 5’-diphosphocholine diacylglycerol; PIS—phosphatidylinositol synthase; PI—phosphatidylinositol; PGPS—phosphatidylglycerolphosphate synthase; PGP—phosphatidylglycerolphosphate; PG—phosphatidylglycerol; CLS—cardiolipin synthase; CL—cardiolipin; PAP—phosphatidic acid phosphatase; CDP—cytidine 5′-diphosphocholine; CEPT—choline/ethanolamine phosphotransferase; DG—diacylglycerol; PE—phosphatidylethanoloamine; PSD 1—phosphatidylserine decarboxylase; PS—phosphatidylserine; PSS—phosphatidylserine synthase; PEMT—phosphatidylethanolamine N-methyltransferase; TG—triacylglycerides; DGAT—diacylglyceride acyltransferase; SM—sphingomyelin; Cer—ceramide; PC—phosphatidylcholine; CPT—phosphatidylcholine; SMS—sphingomyelin synthase.

The levels of the abovementioned metabolites were upregulated in the case of PCa samples. However, a great variance of individual lipid species in the same lipid class was observed (e.g., among the PCs). Phosphatidylcholine is the main phospholipid occurring in eukaryotic cell membranes. In mammalian cells, PC can be de novo synthesized by two independent pathways. One of them is the CDP-choline pathway, which is responsible for the majority of de novo PC biosynthesis in mammalian tissues. The other mechanism of PC production is based on the methylation of PE catalyzed by PE N-methyltransferase. PC synthesized by these pathways is further modified through the Lands cycle remodeling pathway, where fatty acids at the sn-2 position are replaced by the action of phospholipase A2 (iPLA2) and reacylation by lyso-PC acyltransferase. The high variation in the level of metabolites may be explained by an imbalance between the synthesis and remodeling of phospholipids. For this reason, special attention should be paid to the alterations in the expression level of the key enzyme, lysophosphatidylcholine acyltransferase 1 (LPCAT1), in the Lands cycle remodeling pathway [23]. A similar observation was reported by [23] and [24]. Moreover, the authors indicated that the level of phosphocholines increases with the increasing grade of PCa [23]. The main pathways of phospholipid synthesis and modeling are presented in Figure 9 (adapted from Jones et al. ([25]).

The determined increase in the level of phosphocholine is in accordance with the results reported by [8]. The authors indicated the increased concentration of two major structural phospholipids, namely PC and PE, in PCa cells. It can be assumed that this increase in concentration is a consequence of enhanced expression and activity of choline kinase, which is found as an oncogenic factor [8].

Another metabolite identified as statistically significant is LPC. LPC is known as a metabolite which is an substrate known as a dominant metabolite in cellular signaling. LPC plays a significant role in the transport of glycerophospholipids between tissues. It is also involved in the activation of G-protein-coupled receptors, initiates cell proliferation, migration, inflammation, and migration, and is important for cell survival [26,27]. 

SM was also assigned as a metabolite discriminating metabolite between the two tested groups. It is involved in several cellular processes such as cell death and survival. The biological activity of SM is related to cell signaling reactions. This phospholipid is mainly involved in aggregation and fusion. It is also assumed to play a significant role in the development of resistance to chemotherapy [28].

Phospholipids were the topic of interest of the study conducted by Cvetković et al. [5]. The authors noticed that PE is one of the most sensitive plasma phospholipids and may be considered as a specific indicator of disease development and progression (Cvetković et al., 2012). Phospholipids were also determined in urine samples by Min et al. [25]. The authors identified 70 metabolites belonging to the classes of PCs (21), PEs (11), PSs (17), PIs (11), PAs (7), and PGs (3). Based on the statistical analysis, 10 metabolites were assigned as statistically significant. Among them, increased concentrations of PS 18:0/18:1 and PS 16:0/22:6 were observed in the PCa patients, while metabolites such as PS 18:1/18:0, PS 18:0/20:5, PI 18:0/18:1, and PI 16:1/20:2 were downregulated in the patients in comparison to the controls. Moreover, two PS molecules (PS 18:1/18:0 and PS 18:0/20:5) were assigned as the most discriminative. However, the authors indicated that the results were characterized by a relatively large variation in individual concentrations. This observation is in accordance with the results of the present study, and can be explained by the huge biological variability [26]. Taking into account the trend of changes, the results presented by Min et al. were not in accordance with those presented by Cvetković et al. [5], who observed that all groups of phospholipids were decreased in comparison to the control group. Undoubtedly, this was due to the use of a different matrix—urine, which is considered as an organism’s elimination route, and is mostly composed of small and polar metabolites. Moreover, in the study performed by Min et al. [29], only a small set of samples were analyzed. It should also be pointed out that the samples were not matched according to the subjects’ parameters (age, weight).

Additionally, the correlation between the two analyzed methods was evaluated in this study. As observed, the results proved the rightness of the use of the two imaging methods, with each of them being more appropriate and sensitive to different metabolites. The correlation between the data obtained from these techniques was rather non significant, but it should be mentioned that a weak correlation was observed. This proved that the use of the two complementary techniques allowed obtaining a complex picture of the alterations occurring in lipid profiles during disease development or progression.

The lack of correlations between the responses from the individual metabolites detected and confirmed by ESI-QqQ/MS and MALDI-ToF/MS analyses may be due to the use of different protocols for sample preparation. Apart from that, the different physicochemical properties of the metabolites can influence the efficiency of sample ionization, and therefore, detection, depending on the different mechanisms, is associated with the used technique. Determination of qualitative and quantitative correlations within phospholipid “fingerprints” creates the basis for the development of quick and specific diagnostic methods for cancer diseases, including prostate cancer. Continuous development and acquired knowledge give new opportunities in the investigation and validation of new sensitive, diagnostic tests. The presented research gives a new insight for fast and specific screening tests directed towards prostate cancer. The possibility of lipidomic profiling directly from the tissue with the use of imaging methods can also aid the formulation of more accurate diagnosis and prognosis. Potential biomarkers (discriminating features) pointed out by this study can be further investigated in other biological matrices. The determination of these biomarkers may contribute to the selection and monitoring of therapeutic protocols. The proposed techniques may serve as complementary exam, which could be implemented separately or together with bioinformatics approaches in the promotion of timely diagnostics. The prepared statistical models can be implemented to an automated streamline workflow, enabling the generation of a diagnosis report right after processing of analytical data. Such statistical models can be continuously improved by inclusion of expanded learning data. The upload of additional model data can provide outputs containing more specific clinical information.

Apart from this, studies on tissue samples enable to validate the biochemical source of candidate biomarkers reported elsewhere. The comprehensive investigation of changes in lipids metabolism in case of prostate cancer can assist the elucidation of molecular mechanisms associated to this disease, which are so far unknown. A deeper understanding of affected biochemical pathways can serve the development of novel, personalized therapeutic approaches.

## 5. Conclusions

In the case of PCa development, changes in the lipid metabolism lead to the reconfiguration of the existing metabolome. Although several studies have been conducted analyzing the role of lipids in cancer, specific underlying mechanisms are still unknown. Moreover, it remains unclear if the observed metabolic changes are caused by AR reprogramming or if metabolome alteration leads to the reprogramming of the AR activity. For this reason, the present study focused on demonstrating the alterations in lipid synthesis and catabolism in PCa. Two analytical platforms were evaluated, and the results obtained from both approaches indicated that PCa tissues are characterized by modified lipid patterns. A greater number of metabolites and discriminating features were detected by using the ESI-QqQ/MS technique. The response of individual metabolites did not appear to be correlated when considering the two analytical techniques used. However, a notably strong correlation was found between total PC and SM in both data. The classification models developed based on the RF and ANN algorithms were found to be robust and enabled the prediction of the studied cases with a mean accuracy of at least 72%. Superior classifier performance was observed by using ANN for MALDI-ToF/MS data. This suggests that although a lower number of metabolites were detected by this type of analysis, these configured as quite characteristic patterns, serving to discriminate lipidomic profiles into controls and PCa cases.

Nevertheless, in the further stages of the study, several limitations should be considered. Undoubtedly, the study should be performed on a larger population of patients. Different stages of the disease should be taken into account. Secondly, obtained results should be validated by using other biological matrices. Finally, lipidomic metabolism and synthesis, which appears to play a crucial role in in CaP development, should be studied more carefully.

Undoubtedly, further multidisciplinary investigations should be carried out focusing on several factors in order to discover the unknown mechanisms, which could improve the diagnosis and treatment of PCa.

## Figures and Tables

**Table 1:** Characteristic of samples obtained from prostate cancer patients.

Case No.	PSA Level [ng/mL]	Gleason
1	0.008	7
2	0.44	10
3	3.85	9
4	4.10	6
5	4.18	7
6	4.69	6
7	4.70	6
8	4.90	6
9	5.00	7
10	5.01	6
11	5.90	8
12	6.80	7
13	6.80	7
14	7.00	7
15	7.47	7
16	7.50	7
17	8.00	6
18	8.00	9
19	8.26	6
20	8.29	9
21	8.69	6
22	9.00	8
23	9.30	7
24	10.40	7
25	11.00	6
26	13.00	9
27	15.00	7
28	15.30	7
29	20.90	9
30	22.09	6
31	26.00	9
32	27.00	7
33	28.90	6
34	30.82	9
35	31.00	7
36	34.67	9
37	37.00	8
38	42.36	9
39	43.90	8
40	45.04	9

**Table 2:** Information regarding the performance of the developed machine learning models (RF—random forest, ANN—artificial neural network, AUC—area under the curve).

Model		Statistics	PCa vs. Control
RF	ESI-QqQ/MS	Sensitivity	74.20%
		Specificity	84.50%
		AUC	0.866
		Calculated overall accuracy	76.4% (training set)
			80.3% (validation step)
	MALDI-ToF/MS	Sensitivity	64.50%
		Specificity	89.00%
		AUC	0.838
		Calculated overall accuracy	75.0% (training set)
			79.0% (validation step)
ANN	ESI-QqQ/MS	Sensitivity	91.10%
		Specificity	64.50%
		AUC	0.810
		Calculated overall accuracy	80.2% (training set)
			72.5% (validation step)
	MALDI-ToF/MS	Sensitivity	82.20%
		Specificity	96.80%
		AUC	0.960
		Calculated overall accuracy	92.5% (training set)
			87.5% (validation step)

**Table 3:** Compounds assigned as metabolites with discriminant power determined using the acknowledge method.

Compound	Statistical Method		
	Volcano Plot	RF	ANN
LPC 16:1		+	+
LPC 17:0			+
LPC 18:0		+	
LPC 18:1			+
LPC 18:3		+	+
LPC 20:3		+	+
LPC 20:4		+	+
PC 16:0/14:0	+		
PC 16:0/14:1		+	+
PC 16:0/16:0			+
PC 16:0/16:1	+	+	+
PC 16:0/18:0	+	+	
PC 16:0/18:1	+		+
PC 16:0/18:2	+	+	+
PC 16:0/22:6			+
PC 18:0/18:1	+	+	
PC 18:0/22:5	+	+	+
PC 18:1/18:2	+	+	+
PC 18:1/18:1	+		
PC 18:1/20:0	+	+	+
PC 18:1/20:1	+	+	
PC 18:1/20:2	+	+	
PC 18:1/20:3		+	
PC 18:1/20:4	+	+	+
PE 7:0/7:0			+
SM d18:1/16:1			+
SM d18:1/24:0	+	+	+
SM d18:1/24:1		+	
SM d18:1/24:2	+		

## Data Availability

The data presented in this study are available on request from the corresponding author.

## Supplementary Materials

The following are available online at https://www.mdpi.com/article/10.3390/cancers13092000/s1. Table S1: MRM analysis for identification of phospholipids.
