# Supplementary material for: Lipidomics as a Diagnostic Tool for Prostate Cancer
Source: Cancers (Basel). 2021 Apr 21;13(9):2000. doi: 10.3390/cancers13092000 (PMC8122642; doi:10.3390/cancers13092000)
Supplement: Supplementary file 1 [file cancers-13-02000-s001.zip › cancers-1165892-supplementary.pdf]

Table 1. MRM analysis for identification of phospholipids.

Phospholipid	Precursor ion *m/z*	Product ion *m/z*	Polarization mode	Collision energy [V]	Fatty acids
**LPC**	468.0	184.1	Positive	−35	14:0
	496.3	184.1	Positive	−35	16:0
	494.2	184.1	Positive	−35	16:1
	508.0	184.1	Positive	−35	17:1
	510.4	184.1	Positive	−35	17:0
	518.3	184.1	Positive	−35	18:3
	520.0	184.1	Positive	−35	18:2
	522.4	184.1	Positive	−35	18:1
	524.3	184.1	Positive	−35	18:0
	542.2	184.1	Positive	−35	20:5
	544.3	184.1	Positive	−35	20:4
	546.4	184.1	Positive	−35	20:3
	548.3	184.1	Positive	−35	20:2
	550.5	184.1	Positive	−35	20:1
	552.3	184.1	Positive	−35	20:0
	568.3	184.1	Positive	−35	22:6
	600.0	184.1	Positive	−35	24:0
**PC**	730.4	184.1	Positive	−35	16:1/16:1
	732.4	184.1	Positive	−35	16:0/16:1
	734.7	184.1	Positive	−35	16:0/16:0
	746.2	184.1	Positive	−35	15:0/18:1
	758.3	184.1	Positive	−35	16:0/18:2
	760.5	184.1	Positive	−35	16:0/18:1
	762.5	184.1	Positive	−35	16:0/18:0
	766.5	184.1	Positive	−35	15:0/20:5
	774.0	184.1	Positive	−35	16:1/19:0
	780.5	184.1	Positive	−35	18:2/18:3
	782.5	184.1	Positive	−35	16:0/20:4
	784.0	184.1	Positive	−35	18:1/18:2

	786.6	184.1	Positive	−35	16:0/20:2
	788.6	184.1	Positive	−35	18:2/19:0
	790.4	184.1	Positive	−35	18:0/18:0
	796.6	184.1	Positive	−35	17:0/20:4
	800.5	184.1	Positive	−35	18:1/19:1
	802.4	184.1	Positive	−35	18:1/19:0
	804.5	184.1	Positive	−35	16:2/22:5
	806.6	184.1	Positive	−35	16:0/22:6
	808.6	184.1	Positive	−35	18:1/20:4
	810.6	184.1	Positive	−35	18:1/20:3
	812.6	184.1	Positive	−35	18:0/20:3
	814.5	184.1	Positive	−35	18:0/20:2
	816.6	184.1	Positive	−35	18:1/20:0
	818.4	184.1	Positive	−35	18:0/20:0
	824.2	184.1	Positive	−35	18:2/21:2
	828.6	184.1	Positive	−35	18:1/21:1
	830.7	184.1	Positive	−35	18:3/22:5
	832.4	184.1	Positive	−35	18:1/22:6
	834.1	184.1	Positive	−35	18:0/22:6
	836.0	184.1	Positive	−35	18:0/22:5
	838.0	184.1	Positive	−35	20:0/20:4
	840.2	184.1	Positive	−35	20:0/20:3
	842.0	184.1	Positive	−35	20:1/20:1
	844.4	184.1	Positive	−35	20:0/20:1
	848.6	184.1	Positive	−35	20:5/21:0
**SM**	701.1	184.1	Positive	−35	d18:1/16:1
	703.1	184.1	Positive	−35	d18:1/16:0
	717.0	184.1	Positive	−35	d18:1/17:0
	725.0	184.1	Positive	−35	d18:1/18:3
	727.3	184.1	Positive	−35	d18:1/18:2
	731.0	184.1	Positive	−35	d18:1/18:0
	757.3	184.1	Positive	−35	d18:1/20:1
	759.0	184.1	Positive	−35	d18:1/20:0

**LPE**	438.5	196	Negative	35	15:0
	452.5	196	Negative	35	16:0
	466.0	196	Negative	35	17:0
	474.4	196	Negative	35	18:3
	476.0	196	Negative	35	18:2
	478.0	196	Negative	35	18:1
	480.0	196	Negative	35	18:0
	494.0	196	Negative	35	19:0
	500.4	196	Negative	35	20:4
	502.8	196	Negative	35	20:3
	504.4	196	Negative	35	20:2
	506.5	196	Negative	35	20:1
	508.0	196	Negative	35	20:0
	524.0	196	Negative	35	22:6
	526.6	196	Negative	35	22:5
**PE**	690.3	196	Negative	35	16:0/16:0
	710.0	196	Negative	35	16:1/18:3
	714.0	196	Negative	35	16:0/18:2
	716.5	196	Negative	35	16:0/18:1
	718.0	196	Negative	35	16:0/18:0
	738.0	196	Negative	35	16:0/20:4
	740.4	196	Negative	35	18:1/18:2
	742.0	196	Negative	35	18:0/18:2
	744.4	196	Negative	35	18:0/18:1
	746.3	196	Negative	35	18:0/18:0
	760.0	196	Negative	35	18:2/20:5
	762.4	196	Negative	35	16:0/22:6
	764.5	196	Negative	35	18:1/20:4
	766.5	196	Negative	35	18:0/20:4
	768.4	196	Negative	35	18:0/20:3
	772.0	196	Negative	35	18:0/20:1
	770.0	196	Negative	35	18:0/22:0
	790.3	196	Negative	35	18:0/22:6

	792.7	196	Negative	35	18:0/22:5
	794.0	196	Negative	35	20:1/20:3
	796.0	196	Negative	35	18:2/22:1
	798.0	196	Negative	35	18:1/22:1
	824.6	196	Negative	35	20:2/22:1
**LPG**	481.0	171	Negative	35	16:1
	507.1	171	Negative	35	18:2
	509.1	171	Negative	35	18:1
	511.0	171	Negative	35	18:0
**PG**	693.1	171	Negative	35	14:0/16:0
	715.7	171	Negative	35	14:0/18:3
	717.0	171	Negative	35	16:1/16:1
	743.1	171	Negative	35	16:0/18:2
	763.3	171	Negative	35	17:0/18:1
	777.6	171	Negative	35	18:0/18:0
**LPA**	409.0	153	Negative	35	16:0
	437.1	153	Negative	35	18:0
**PA**	591.1	153	Negative	35	14:0/14:0
	671.2	153	Negative	35	16:1/18:1
	673.1	153	Negative	35	16:0/18:1
	689.6	153	Negative	35	15:0/20:0
	699.1	153	Negative	35	16:1/20:1
	725.4	153	Negative	35	18:0/20:3
	727.3	153	Negative	35	18:2/20:0
**LPI**	597.5	241	Negative	35	18:1
	599.4	241	Negative	35	18:0
**PI**	833.6	241	Negative	35	16:0/18:1
